# Acetoacetate Based Thermosets Prepared by Dual-Michael Addition Reactions

**DOI:** 10.3390/polym11091408

**Published:** 2019-08-27

**Authors:** Osman Konuray, Xavier Fernández-Francos, Xavier Ramis, Àngels Serra

**Affiliations:** 1 Thermodynamics Laboratory, ETSEIB, Universitat Politècnica de Catalunya, Av. Diagonal 647, 08028 Barcelona, Spain; 2 Department of Analytical and Organic Chemistry, Universitat Rovira i Virgili, C/Marcel lí Domingo s/n, 43007 Tarragona, Spain

**Keywords:** dual-curing, Michael addition, click chemistry, acetoacetate, divinyl sulfone, acrylate

## Abstract

A novel set of dual-curable multiacetoacetate-multiacrylate-divinyl sulfone ternary materials with versatile and manipulable properties are presented. In contrast to common dual-curing systems, the first stage polymer herein consists of a densely crosslinked, high *T_g_* network as a result of base-catalyzed multiacetoacetate-divinyl sulfone Michael addition. A more flexible secondary network forms after base-catalyzed Michael addition of remaining multiacetoacetate to multiacrylate. Curing is truly sequential as the rates of the two Michael additions are significantly different. Curing kinetics were analyzed using differential scanning calorimetry (DSC) and Fourier-transform infrared (FTIR). The materials at each curing stage were characterized using dynamic mechanical analysis (DMA) and SEM. Although some phase separation was observed in certain formulations, the incompatibilities were minimized when the molar percentage of the acetoacetate-divinyl sulfone polymer network was above 75%. Furthermore, the environmental scanning electron microscopy (ESEM) images of these materials show that the more flexible acetoacetate-acrylate phase is dispersed in the form of polymeric spheres within the rigid acetoacetate-divinyl sulfone matrix. This unique dual microstructure can potentially render these materials highly resilient in applications requiring densely crosslinked polymer architectures with enhanced toughness.

## 1. Introduction

Recently, more and more thermosetting polymers are prepared by versatile dual-curing methodologies due to their many advantages in terms of material processing [1,2,3,4,5]. A dual-curing process is a combination of two compatible and selective polymerizations carried out either simultaneously or sequentially. In contrast, for simultaneous dual-curing, a sequential process facilitates processing flexibility as it affords the storage of stable intermediate (i.e., partially-cured) materials. The properties of these intermediate materials can easily be tailored a priori by careful selection of monomers and their proportions. As a result, they can range from liquid-like adhesives to semi-rigid and conformable solids [6]. The second curing stage is initiated at a later time using a different stimulus to yield the final material with significantly improved mechanical properties.

Click chemistry is an effective tool in designing efficient and environmentally safe dual-curing processes [1,7,8,9]. Among the numerous click polymerization reactions, Michael additions are arguably the most common reaction type in recent dual-curing systems [10,11,12,13]. A Michael addition is described as the conjugate addition of nucleophilic carbanions to activated double bonds [10]. The reaction can be initiated by basic or nucleophilic catalysts. A variety of nucleophiles (Michael donors) and activated double bond compounds (Michael acceptors) are commercially available. In Michael-type dual curing systems, the Michael donor is more frequently a thiol [3,11,12,14], or an amine [4,15,16,17] Acetoacetates are also employed, but less frequently [18,19,20]. As a matter of fact, acetoacetates introduce many advantages to dual-curing systems. Their Michael reaction kinetics are slower compared to other donors therefore no premature curing takes places during sample preparation. Their polymers have a high crosslink density and superior mechanical properties especially if monomeric acetoacetates have high functionalities. Consequently, acetoacetate functional monomers are used in different applications, such as low energy curing coatings and adhesives [10,21,22].

Since it is a novelty on its own, this paper starts with a preliminary kinetic analysis of the acetoacetate-divinyl sulfone Michael addition. Although the general Michael addition mechanism is well documented, this particular Michael donor-acceptor pair has not been studied before. After establishing the conditions for this reaction, this study prepared a set of dual curable multiacetoacetate-multiacrylate-divinyl sulfone ternary formulations with versatile and manipulable properties. Depending on the initial monomer mixture, (after a first curing stage) liquid or solid intermediate materials can be obtained which can be used immediately in the application or can be transported and/or stored for prolonged periods. This processing flexibility can be desirable for demanding applications. In contrast to common dual-curing systems, the first stage polymer herein consists of a densely crosslinked, high *T_g_* network as a result of the base-catalyzed multiacetoacetate-divinyl sulfone Michael addition. A more flexible secondary network forms after a base-catalyzed Michael addition of remaining multiacetoacetate to multiacrylate. This is not surprising as it was reported by other researchers that, compared to acrylic monomers, vinyl sulfone based monomers yield high *T_g_* materials due to structural effects, π-electronic and dipolar interactions and hydrogen bonding [23]. To obtain a truly sequential curing process, the authors took advantage of the intrinsic difference of the Michael reaction kinetics of acrylates and vinyl sulfones. This bestows selectivity to the acetoacetate-vinyl sulfone reaction in any mixture in which acrylate groups are also present, as was documented in the past [12,24,25]. A phase separation is observed in these materials induced by the polymerization process. However, the study shows that the extent of the incompatibilization can be controlled by changing the monomer feed ratio. The microstructure of the resulting dual-network materials suggests that the second stage multiacetate-multiacrylate network might potentially act as a toughness enhancer.

## 2. Materials and Methods

A trifunctional acetoacetate K-FLEX^®^7301 (hereafter referred to as KF, equivalent weight per active hydrogen of 125 g/e according to the data sheet), supplied by King Industries, Norwalk, CT, USA, was used as a Michael reagent donor. Michael acceptors, divinyl sulfone (DVS, 118.15 g/mol) and hexanediol diacrylate (HDDA, 226.27 g·mol^−1^), supplied by Sigma Aldrich, Saint Louis, MO, were used without any purification. The catalyst 1-methylimidazole (1-MI), a weak base, was also supplied by Sigma Aldrich and was used without purification. The structures of all chemicals used are given in Table 1.

**Scheme 1:**
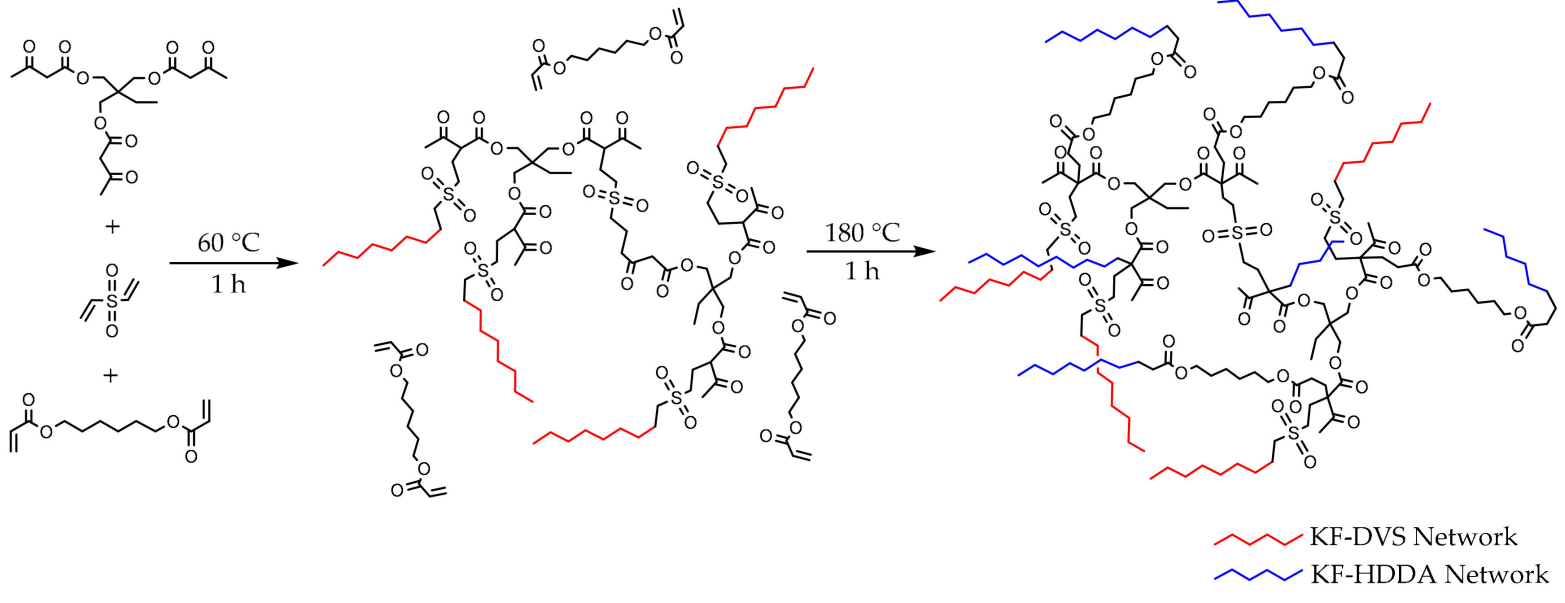
The curing process used to produce the KF- divinyl sulfone (DVS)- hexanediol diacrylate (HDDA) dual network.

The stoichiometric KF-DVS formulations with 2 phr of 1MI were prepared, assuming that the functionality (*f*) of KF was 6 or 3. The stoichiometric dual formulations containing different KF:DVS:HDDA ratios were also prepared, but it was assumed that *f* = 6. The dual-curing process is depicted in Scheme 1. The notation and composition of the different dual formulations are shown in Table 2.

In the preliminary analysis of the KF-DVS reaction kinetics, differential scanning calorimetry (DSC) was performed under isothermal conditions and by selecting adequate temperatures. The runs were continued until no further heat release was observed and the maximal double bond conversions (*x*) were calculated using Equation (1).

(1) $$x=1-\frac{∆H_{residual}}{∆H_{total}}$$

where $∆H_{total}$ was determined in a dynamic scan from 30 to 250 °C at a heating rate of 10 °C/min. The glass transition temperatures, $T_{g},$ of the samples were determined as the halfway point in the heat capacity step during a dynamic DSC scan at 10 °C min^−1^. In order to model the dependence of the $T_{g}$ on the degree of conversion, $T_{g}\left(x\right)$, the expression derived by Venditti and Gillham [26] has been used:

(2) $$lnT_{g}\left(x\right)=\frac{\left(1-x\right)\cdot lnT_{g0}+\lambda \cdot x\cdot lnT_{g\infty }}{\left(1-x\right)+\lambda \cdot x}$$

(3) $$\lambda =\frac{∆C_{p\infty }}{∆C_{p0}}$$

where $T_{g0}$ and $T_{g\infty }$ are the glass transition temperatures (in K) of the uncured and crosslinked materials, and $∆C_{p0}$ and $∆C_{p\infty }$ are the increase in heat capacity during the glass transition of the uncured and crosslinked materials, respectively, measured in the same DSC experiment.

To further study the curing kinetics, a Brucker Vertex 70 Fourier-transform infrared (FTIR) spectrometer equipped with an attenuated total refection (ATR) accessory (Golden gateTM, Specac Ltd., Orpington, UK) which is temperature controlled (heated single-reflection diamond ATR crystal) was used to monitor the evolution of the reactive groups. Real-time spectra were collected at the chosen curing temperatures in absorbance mode with a resolution of 4 cm^−1^ and a wavelength range from 400 to 4000 cm^−1^, averaging 20 scans for each spectrum. The bands at 700 and 775 cm^−1^ [24] were used to monitor the evolution of vinyl sulfone double bonds, whereas the band at 810 cm^−1^ [27] was used to monitor acrylate double bonds disappearance. The peaks were normalized using the carbonyl ester band at 1720 cm^−1^ [28]. The conversion of double bonds is denoted as *x* and it is defined by Equation (4).

(4) $$x=1-\frac{A_{t}{}^{\prime }}{A_{0}{}^{\prime }}$$

where $A^{\prime }$ is the normalized area of the acrylate or vinyl sulfone bands, and the subscripts *t* and *0* indicate the curing time and the beginning of the curing, respectively.

Final materials, along with intermediate materials which could be obtained in solid form, were analyzed using a dynamic mechanical analysis (DMA). A TA Instruments DMA Q800 device was used. The test specimens were prepared by pouring the liquid monomer mixtures in prismatic molds of dimensions ca. 1 × 13 × 20 mm^3^ and oven curing: 1 h at 60 °C (curing stage 1, KF-DVS reaction) followed by 1 h at 180 °C (curing stage 2, KF-HDDA reaction), and a 30 min. postcuring at 200 °C to ensure complete conversion. The choice of curing temperatures is based on the kinetic analyses which is explained in the following sections. The prepared specimens were analyzed using a single cantilever clamp at a frequency of 1 Hz and 0.05% strain at 3 °C/min within adequately chosen temperature ranges to observe a complete network relaxation of each sample. The peak temperatures of tan *δ* curves were taken as *α*-relaxation temperatures. The peak temperatures of loss moduli curves were taken as approximation of thermal *T_g_*.

Thermogravimetric analysis (TGA) was carried out with a Mettler TGA/SDTA 851e/LF/1100 thermobalance. The fully cured samples with an approximate mass of 10 mg were thermally degraded between 30 and 800 °C at a heating rate of 10 °C/min in a nitrogen atmosphere (50 cm^3^/min measured under normal conditions).

The environmental scanning electron microscopy (ESEM) was used to examine the fracture surfaces of the materials prepared. A Quanta 600 environmental scanning electron microscopy (FEI Company, Hillsboro, Oregon, USA) allowed collecting micrographs at 10–20 kV and at a low vacuum mode without the need to coat the samples.

## 3. Results and Discussion

### 3.1. KF-DVS Pure System (Stage 1)

Firstly, the neat multiacetoacetate (KF)–divinyl sulfone (DVS) system (*DVS-HDDA 1-0*) was characterized with respect to reaction kinetics. The choice of a KF-DVS molar ratio depends on the desired effective functionality (*f*) of KF. If only the primary acetoacetate hydrogens are assumed to undergo a Michael addition, functionality is 3 (*f* = 3). Whereas if both primary and secondary acetoacetate hydrogens are assumed to react, functionality becomes 6 (*f* = 6). Preliminarily, both cases were tested. *DVS-HDDA 1-0* formulations were prepared and a Michael addition carried out at 35 and 80 °C in the presence of 0.5% (*w*/*w*) of 1-methylimidazole (1-MI). In Table 3, the final conversions reached in isothermal conditions (calculated using Equation (1)) and the time to reach these conversions are given with regard to assumed functionalities.

**Figure 1:**
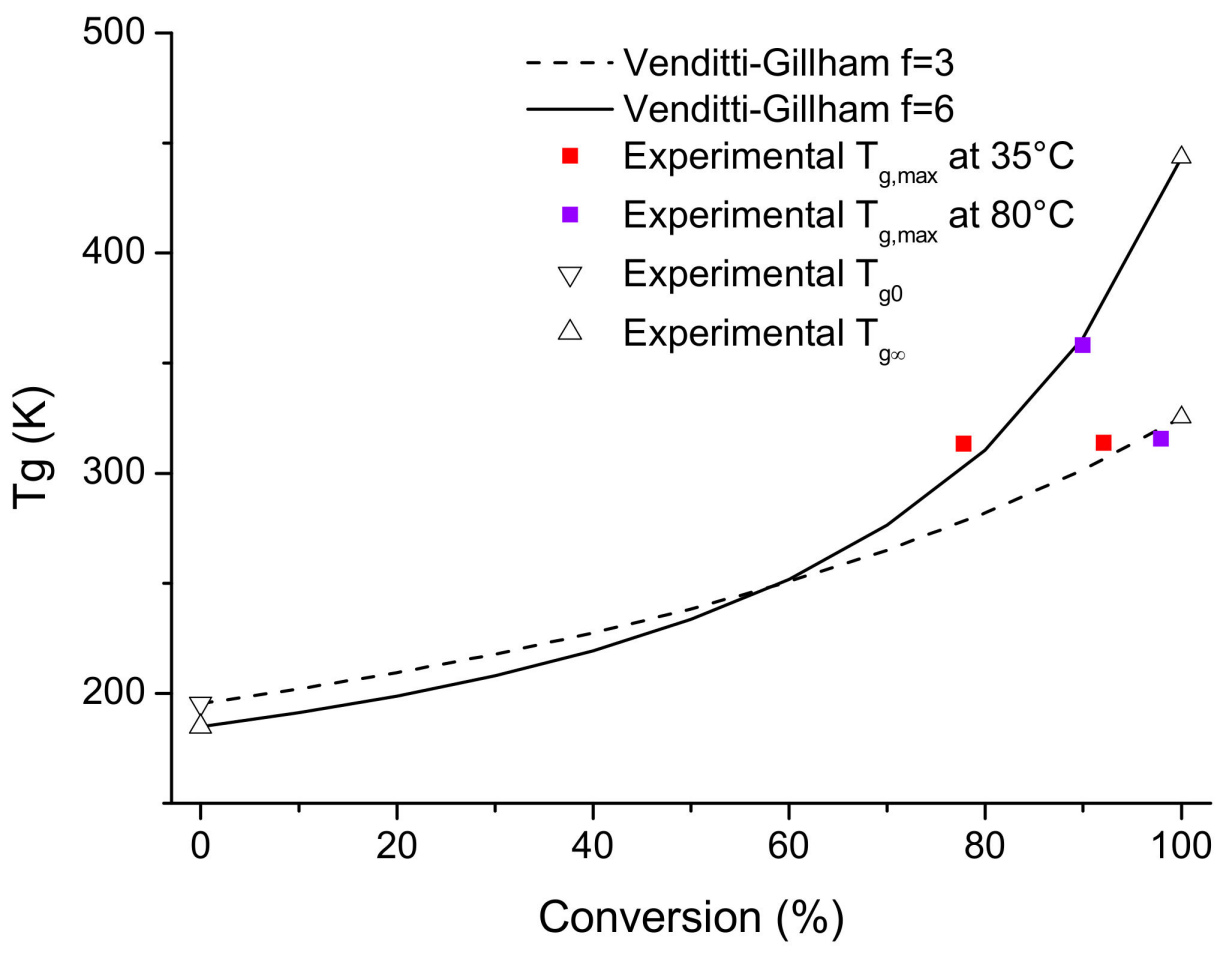
The relationship between *T_g_* and conversion for the KF-DVS pure system (*DVS-HDDA 1-0*). Experimental *T_g_* were determined by DSC. *T_g0_* and *T_g__∞_* were used to construct the Venditti-Gillham curves. The experimentally determined maximal *T_g_* of formulations with different acetoacetate functionalities (colored squares) fit well to their corresponding curves.

In Figure 1, the accuracy of the Venditti and Gillham approximation for the *T_g_*-*x* relationship is demonstrated. The thermal parameters determined by DSC and used in the Equation (2) are tabulated in Table 4. As can be seen, vitrification and therefore incomplete conversion took place in all the experiments with *f* = 6 because *T_g_*_∞_ was higher than the curing temperature. For *f* = 3, vitrification was only observed at the curing temperature of 35 °C, and conversion at 80 °C was not complete because of the slow reaction kinetics at the end of the curing process. Higher conversions were achieved when *f* = 3 due to the lower value of *T_g_*_∞_ (see Table 4). In contrast, for *f* = 6, the lower conversion is achieved because of the significantly higher value of *T_g_*_∞_. The low value of *λ* for *f* = 6 (Table 4) is an indication of the significant reduction in mobility that takes place during the crosslinking process because of the densely crosslinked structure that is being formed, but at the same time it implies that *T_g_* is a weak function of *x* up to high values of *x*. As a result, even for *f* = 6, vitrification is not a significant impediment to reach high conversions. In the light of these findings, *f* was fixed at 6 for the dual formulations prepared in the rest of the study. Furthermore, the Stage 1 curing temperature was chosen as 60 °C. As is shown later, within the dual formulation, the KF-DVS reaction proceeds to completion in 1 h at this temperature since dilution by the KF-HDDA part overcomes any mobility restriction.

### 3.2. Dual System KF-DVS-HDDA (2:1:1 Molar Ratio)

**Figure 2:**
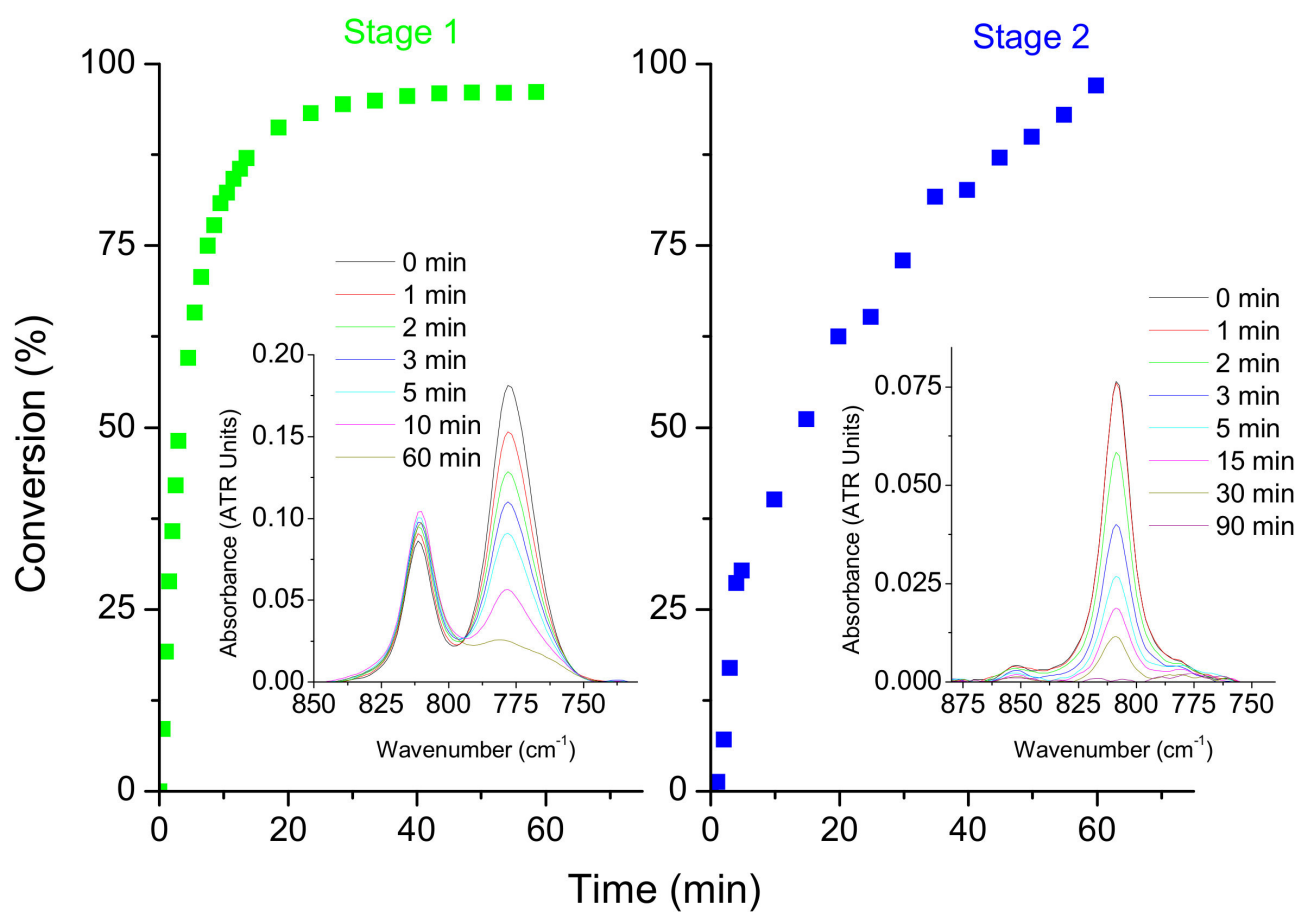
The Fourier-transform infrared (FTIR) double bond conversion for the dual-curing of *DVS-HDDA 1-1*. Note that the absorption peak at 810 cm^−1^ (inset figure on the left) remains intact during stage 1, confirming the selectivity of the curing reaction. Curing temperatures are 60 °C and 180 °C for stages 1 and 2, respectively.

To study the overall curing kinetics, the formulation *DVS-HDDA 1-1* was chosen. FTIR was used to follow the evolution of the different species during both stages. Within the dual formulation, the first stage KF-DVS curing reaction virtually consumes all DVS monomers, in contrast with the pure KF-DVS system, even at a lower temperature (see Figure 2). As postulated previously, the KF excess and HDDA provide the convenient dilution to overcome mobility restrictions posed during the formation of the KF-DVS network. No acrylate reaction was observed in this first curing stage, as could be expected, given the better Michael acceptor character of the vinylsulfone groups in comparison with acrylate groups [24] (see inset in Figure 2, stage 1). The second stage, the KF-HDDA curing reaction, was carried out at 180 °C, with a comparable rate to stage 1. To ensure conversion of all monomers, a postcuring was performed for 30 min at 200 °C. Similar kinetics were observed in all remaining formulations whose results are not shown here for space considerations. Given the selectivity in the first curing stage and the significant difference between curing temperatures at stages 1 and 2, it can be safely stated that ternary KF-DVS-HDDA formulations are truly sequential dual-curing systems.

### 3.3. Material Properties

**Figure 3:**
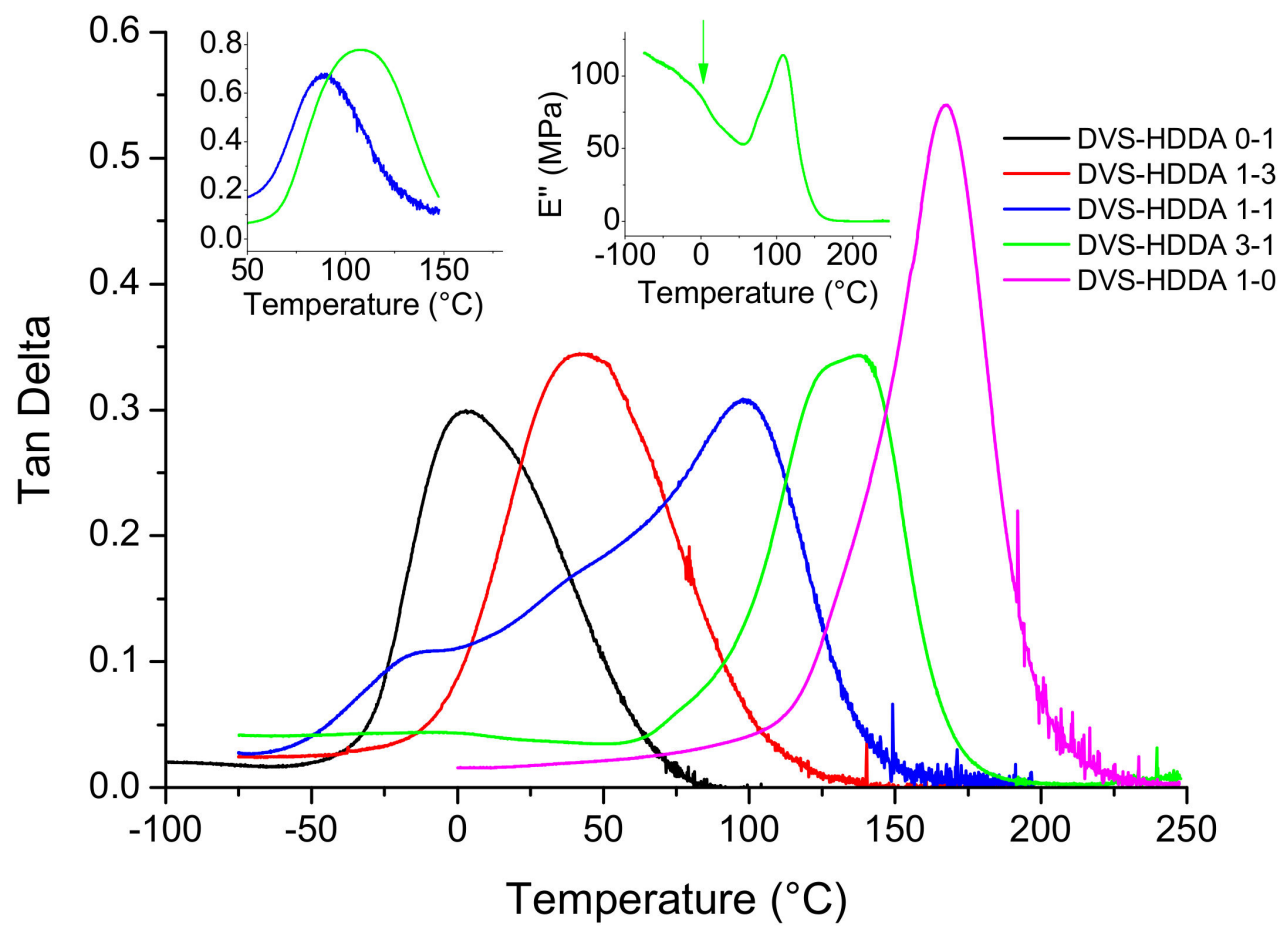
α-relaxations of KF-DVS-HDDA ternary materials. The left-hand side inset shows α-relaxation profiles of the intermediate materials which were solid and thus a dynamic mechanical analysis (DMA) analysis was possible. The loss modulus curve of DVS-HDDA 3-1 is shown in the right-hand side inset in which a stronger peak is produced by the acrylate-rich phase.

The thermal-mechanical properties of intermediate and fully-cured dual formulations were analyzed with DMA and DSC. The α-relaxation curves of fully cured formulations determined with DMA are given in Figure 3. As can be seen, in all dual formulations, α-relaxations become somewhat multi-modal, indicating network heterogeneity, especially for the dual-curing formulation *DVS-HDDA 1-1*. In fact, formulation *DVS-HDDA 1-1* and *DVS-HDDA 3-1* had opaque appearances, indicating phase separation, while *DVS-HDDA 1-3* was transparent, indicating that the material was homogeneous. The phase separation is not perfect, as the tan δ peaks of dual formulations did not correspond exactly to those of the constituent parts (i.e., Neat KF-DVS and KF-HDDA). Furthermore, at the end of the Stage 1 of the dual curing, the *DVS-HDDA 1-1* and *DVS-HDDA 3-1* materials (which are DVS-rich) had already exhibited polymerization-induced phase separation due to the incompatibility between the KF-DVS network formed during Stage 1 and the unreacted monomers (the excess KF and HDDA). This phenomenon was observed previously for epoxy-acrylic systems [29,30]. Although the acrylate-rich phase in DVS-HDDA 3-1 only produced a slightly detectable peak in its tan delta, it produced a stronger one in its loss modulus at approximately 0 °C (See Figure 3, right-hand side inset).

**Figure 4:**
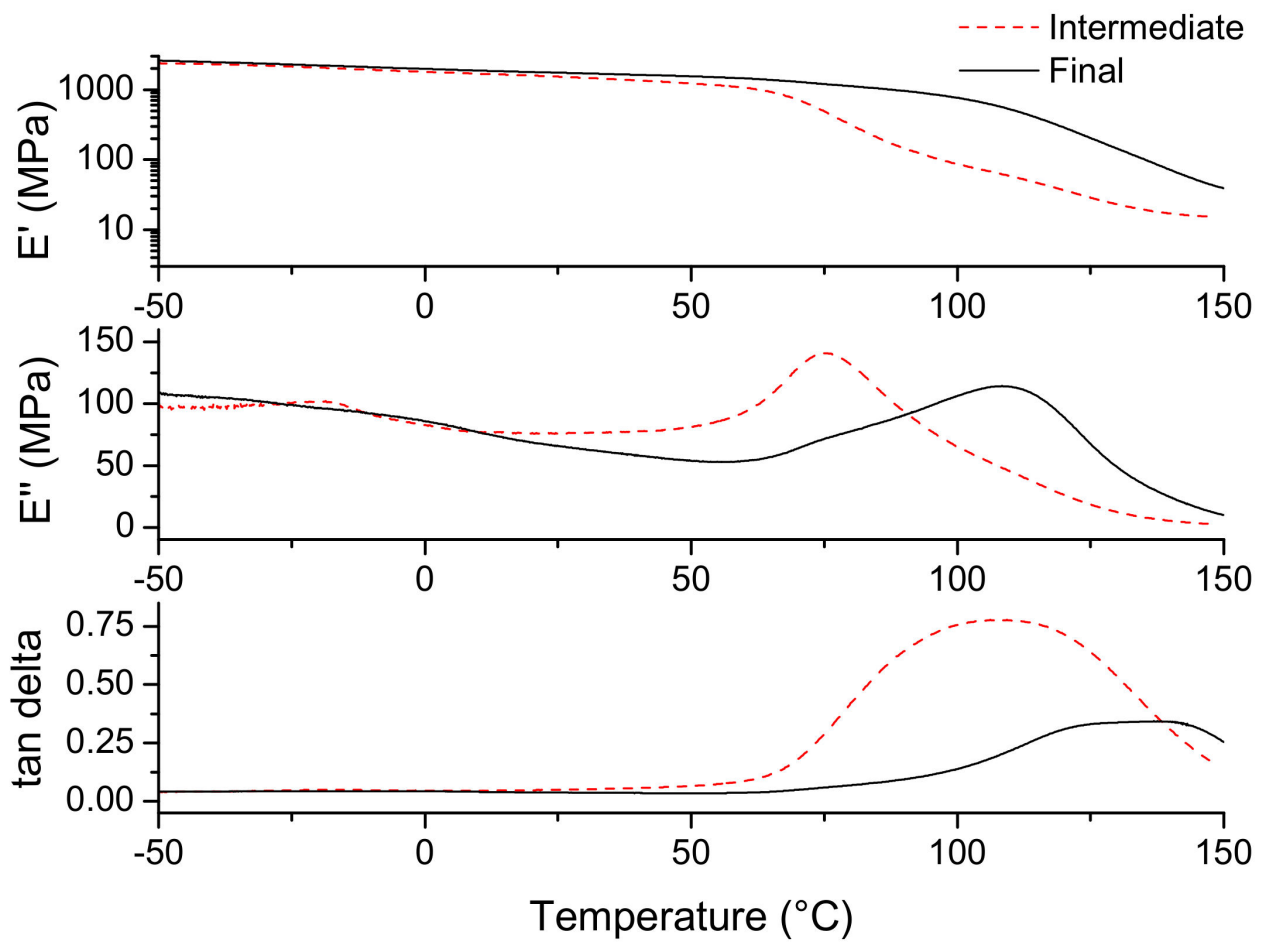
Storage moduli (*E*’), loss moduli (*E*”) and tan δ curves of DVS-HDDA 3-1 intermediate (dashed red) and final (solid black) materials.

The evolution of the viscoelastic properties of the *DVS-HDDA-3-1* formulation is shown in Figure 4. As a matter of fact, the material can be thought of as a KF-DVS system covalently modified with 25% (by moles) of a KF-HDDA network. Since monomer HDDA had a significantly more flexible backbone in comparison to DVS, the increase in *T_g_* after the KF-HDDA reaction (curing stage 2) was slight. A closer look at the evolution of the loss modulus *E*” hints at the presence of a secondary relaxation in the intermediate material at approximately −20 °C, which disappears in the fully cured material. This suggests the presence of a second phase in the intermediate material, as discussed above.

**Figure 5:**
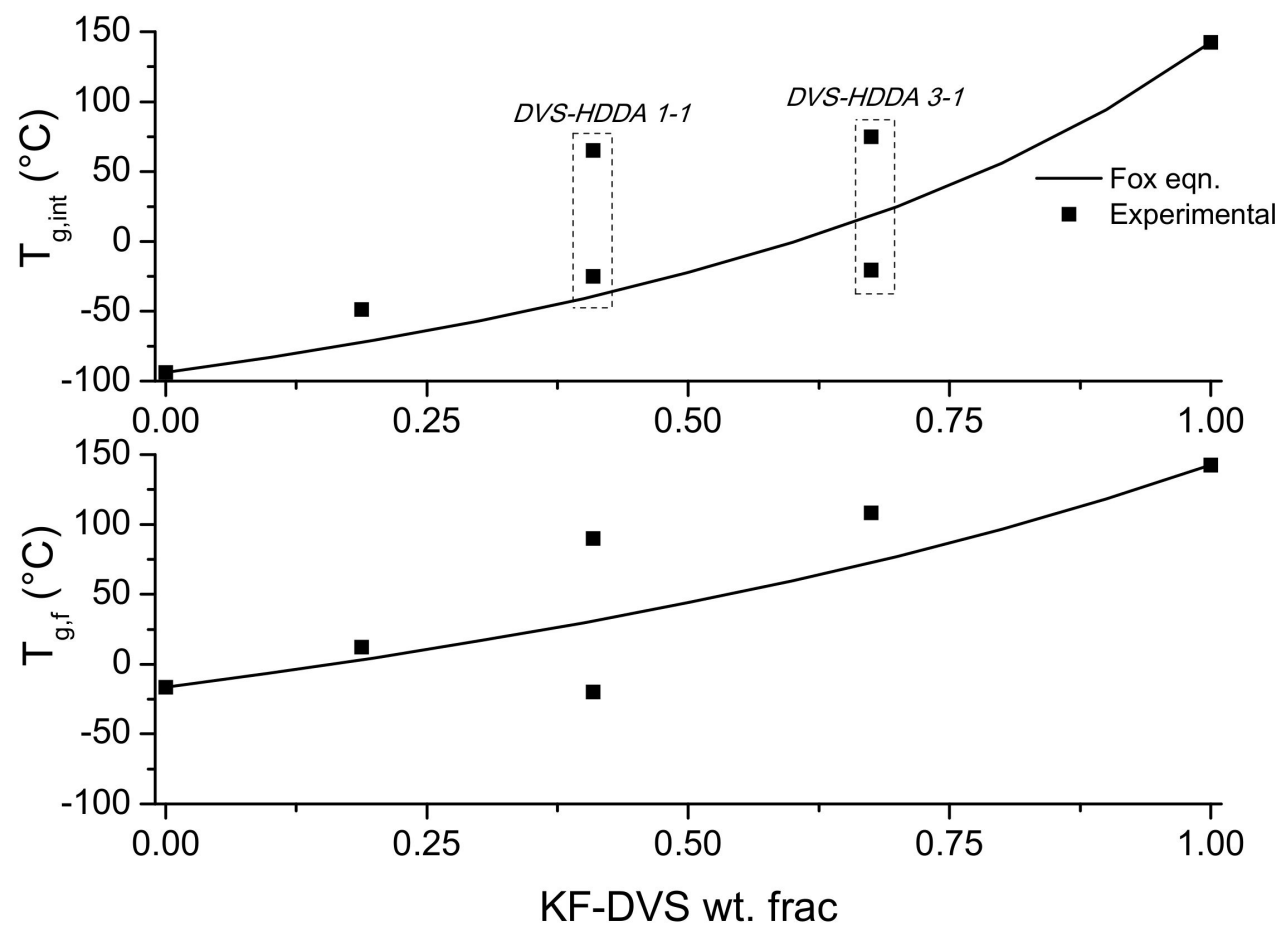
Intermediate (**top**) and final *T_g_* (**bottom**) as a function of DVS weight fraction. The curves are drawn using the Fox equation [31]. The filled squares represent experimentally determined values.

As further analysis, the temperatures at the maximum loss modulus which are empirically correlated to the *T_g_* determined by DSC were plotted together with the Fox approximations [31] as a function of DVS weight fraction. This study determined the theoretical values of the intermediate *T_g,int_* making use of the *T_g,f_* of the fully cured *DVS-HDDA 1-0* material (see Table 4) and the *T_g_* of the uncured *DVS-HDDA 0-1* material, which was of −94 °C. For the final *T_g,f_* the *T_g,f_* of the fully cured *DVS-HDDA 1-0* material (see Table 4) and the *T_g_* of the fully cured *DVS-HDDA 0-1* was used, which had a value of −17 °C. The resulting *T_g,int_* and *T_g,f_* graphs are given in Figure 5. Conventionally, thermal *T_g_s* (obtained by DSC) of the intermediate and final dual-curing should have been compared with predictions made using the Fox approximation. However, the materials used in this study did not exhibit clear glass transitions in the DSC, possibly due to their highly heterogeneous polymer networks. At the intermediate stage, *DVS-HDDA 1-1* and *DVS-HDDA 3-1* had to be analyzed with DMA as they had attained a gel state, while intermediate *DVS-HDDA 3-1*, which was ungelled, was analyzed with DSC. The final materials were all characterized with DMA. In order to make meaningful comparisons, the values of *T_g_* were assigned, making use of the peaks of the loss modulus *E*” curves, which should give values lower than *tan δ* peak temperatures and closer to calorimetric *T_g_*. Given the breadth of the glass transitions, in some cases, the position of the relaxation had to be assigned taking also into consideration *E*’ and *tan δ* curves.

For the *DVS-HDDA 1-1* material, two clearly separated *E*” peaks were observed at both curing stages. As a matter of fact, the phase separation was visually evident also in the fully cured specimens of this formulation. However, had the phase separation been perfect, each peak would have coincided to its constituting part (KF-DVS or KF-HDDA). The two relaxation temperatures of the DVS-HDDA 1-1 final material suggest that one phase is practically KF-HDDA. As can be seen in Figure 5, the lower *T_gf_* of *DVS-HDDA 1-1* is virtually equal to the *T_gf_* of *DVS-HDDA 0-1* (neat KF-HDDA). On the other hand, the higher *T_g_* phase is predominantly KF-DVS with a small content of KF-HDDA. This causes the second *T_gf_* to decrease slightly from the *T_gf_* of *DVS-HDDA 1-0* (neat KF-DVS). Noticeably, the *T_g_* of the lower *T_g_* phase does not increase upon the second stage reaction, indicating that this phase might contain a fraction of unreacted HDDA or KF monomers. The *DVS-HDDA 3-1* intermediate material also exhibited two *E*” peaks, the lower being less evident. Notably, the intermediate *DVS-HDDA 1-1* and *DVS-HDDA 3-1* materials have similar relaxation temperatures. Again, this is due to the phase separation. Upon stage 2 curing of the *DVS-HDDA 3-1* material, the relaxation temperature increased significantly (up to ca. 120 °C) and the relaxation profile became apparently unimodal (Figure 5, bottom graph). However, the fully cured material was opaque, suggesting there was some phase separation. If the lower *T_g_* (associated to the phase rich in KF-HDDA network) had produced a *E*” peak with sufficient intensity to allow measurement, this would also allow the calculation of an average *T_g_* for the DVS-HDDA 3-1 final material and it would lie significantly closer to the Fox curve. In some materials, the *T_g_* determination of the KF-HDDA rich phase was not as precise as in others due to the complex behavior of *E*” and *tan δ*. These required cross-referencing of *E*” peaks with *E*’ drops, since some of the *E*” peaks were not accompanied by any significant drop in *E*’ and therefore had to be discarded.

**Figure 6:**
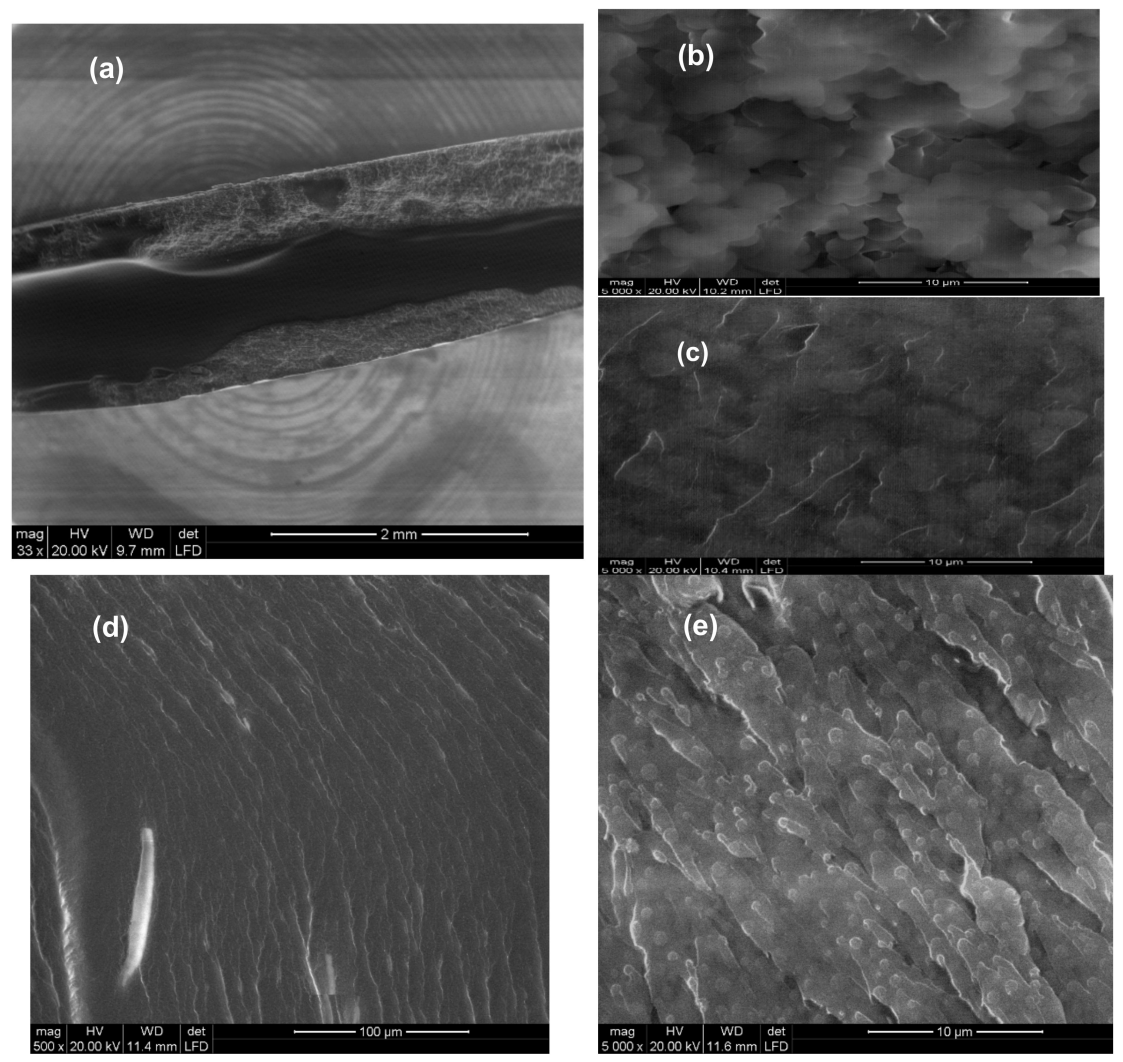
SEM images of final stage materials at low and high magnifications (left and right-hand side images, respectively). At the millimeter scale, the DVS-HDDA 1-1 material exhibits a dramatic phase separation (**a**) with a darker and denser interior phase (**b**) and a porous exterior phase with a lighter color (**c**). The DVS-HDDA 3-1 material appears more homogeneous at a scale of 100 microns (**d**). Further magnification reveals the disperse formation of spheres (prominently composed of the KF-HDDA phase) within the continuous KF-DVS phase (**e**).

In order to elucidate the morphology of the fully-cured materials, ESEM was performed on the fractured surfaces of the specimens of different formulations. The results of the analysis are shown in Figure 6. Given the results from the DMA analysis, the phase separation should be more evident in *DVS-HDDA 1-1*. This hypothesis was confirmed by ESEM analysis shown in Figure 6a. The KF-HDDA (interior) phase seems to be sandwiched between the two layers of KF-DVS (exterior) network. It is hypothesized that upon stage 1 curing, an incompatible KF-DVS polymeric phase came about in which a small content of uncured HDDA was trapped. For both the intermediate and final *DVS-HDDA 1-1* materials, this HDDA content caused the higher of the two *T_g_* to deviate from a pure KF-DVS network. (See Figure 3, blue curves; and Figure 5). SEM pictures evidenced a strong incompatibilization between the HDDA-rich and DVS-rich phases.

However, the ESEM images of *DVS-HDDA 3-1* suggest no such incompatibilization (Figure 6b, left-hand side). An acrylate-rich phase seems to have developed in the form of spheres which are covalently bound to the continuous KF-DVS matrix (Figure 6b right-hand side images). As further confirmation, another formulation was prepared with a KF:DVS:HDDA molar ratio of 1:0.88:0.12 (formulation coded as *DVS-HDDA 7-1*), its *T_g_* was measured in DMA, and its ESEM images taken. The number of phase-separated spheres decreased with decreasing HDDA content, confirming the above hypothesis (See Figure S1 in Supporting Information). It is argued that this particular structure would impart improved toughness to the final material as long as the amount of the KF-HDDA phase is limited to avoid massive incompatibilization as was the case in *DVS-HDDA 1-1* [32]. As can be seen in Figure 6b, the sizes of these covalently bound spheres might be sufficiently large to attenuate or even stop crack propagation. In this way, the impact energy can be dissipated.

### 3.4. Thermal Degradation of Materials

**Figure 7:**
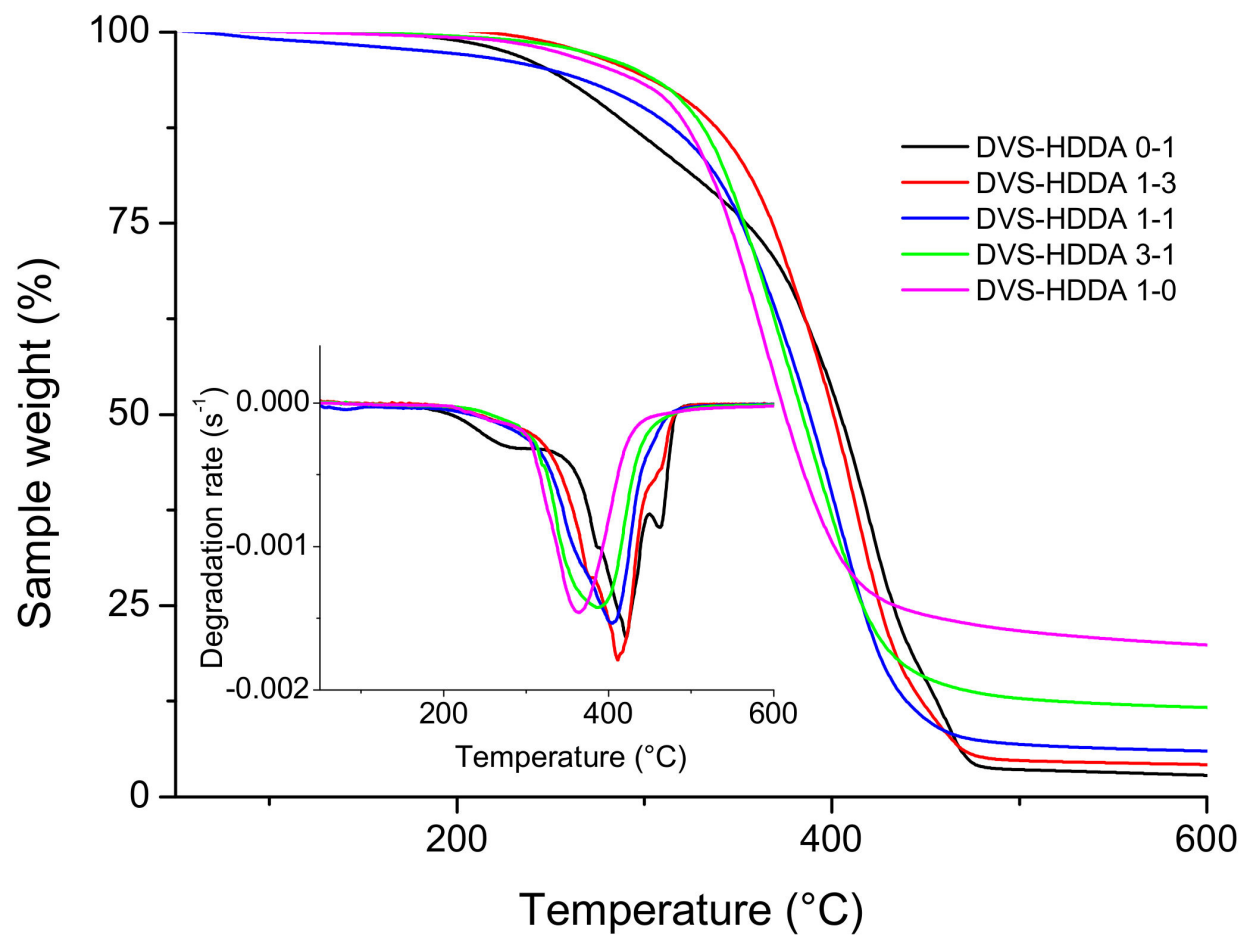
Thermogravimetric analysis (TGA) weight loss curves and the thermal degradation rates (inset) of the final stage materials.

In Figure 7, the TGA curves of the final materials are given together with the degradation rates (inset). When *DVS-HDDA 1-0* and *DVS-HDDA 0-1* are compared, the early onset of degradation of the latter material can be appreciated. This can be explained by its lower crosslinking density (recall that the functionality of both Michael acceptors DVS and HDDA are the same, but HDDA is a larger molecule with a long aliphatic chain). However, there is no clear explanation for the thermal degradation profiles of the dual materials used in this study. As per the authors’ ongoing discussion about the distinct structure of these dual materials, the two constitutent networks are covalently interacting. This complicates the dissection of their individual effects on the thermal degradation behavior. Noticeably, the degradation of *DVS-HDDA-1-1* starts earlier, suggesting the presence of unreacted monomers in the fully cured material, as suggested by the previous analysis of the *T_g_* of intermediate and the final materials. This could be due to the strong incompatibilization between HDDA-rich and DVS-rich phases. Nevertheless, the char yields clearly show an increasing trend, with increasing DVS content (the plateau above 450 °C).

## 4. Conclusions

A set of dual curable multiacetoacetate-multiacrylate-divinyl sulfone ternary formulations were prepared which attained a wide range of physical and thermomechanical properties. The curing process is truly sequential due to the significant difference between the reaction rates of the two curing stages. The first curing stage was a Michael addition of a proton donating multiacetoacetate to a divinyl sulfone. The kinetics of this relatively less studied Michael addition pair was analyzed and it was confirmed that both acetoacetate hydrogens were able to react at a moderate temperature and rate. The densely crosslinked first stage multiacetoacetate-divinyl sulfone polymer network gave the final materials high rigidity, whereas the second stage multiacetoacetate-diacrylate second stage networks introduced flexibility. The dual materials exhibited phase separation due to the incompatibility of the two polymer networks at compositions with the DVS molar ratios equal to or higher than 50%. Nevertheless, the final material with 75% molar ratio of acetoacetate-divinyl sulfone developed a desirable microstructure, with a *T_g_* comparable to its neat counterpart. As further research, a formal comparison of these novel materials with epoxy and/or acrylate based thermosets with respect to thermomechanical and tensile properties could be undertaken.

## Figures and Tables

**Table 1:** Monomers and the catalyst used in the work.

Monomer Chemical Structure	Abbreviation
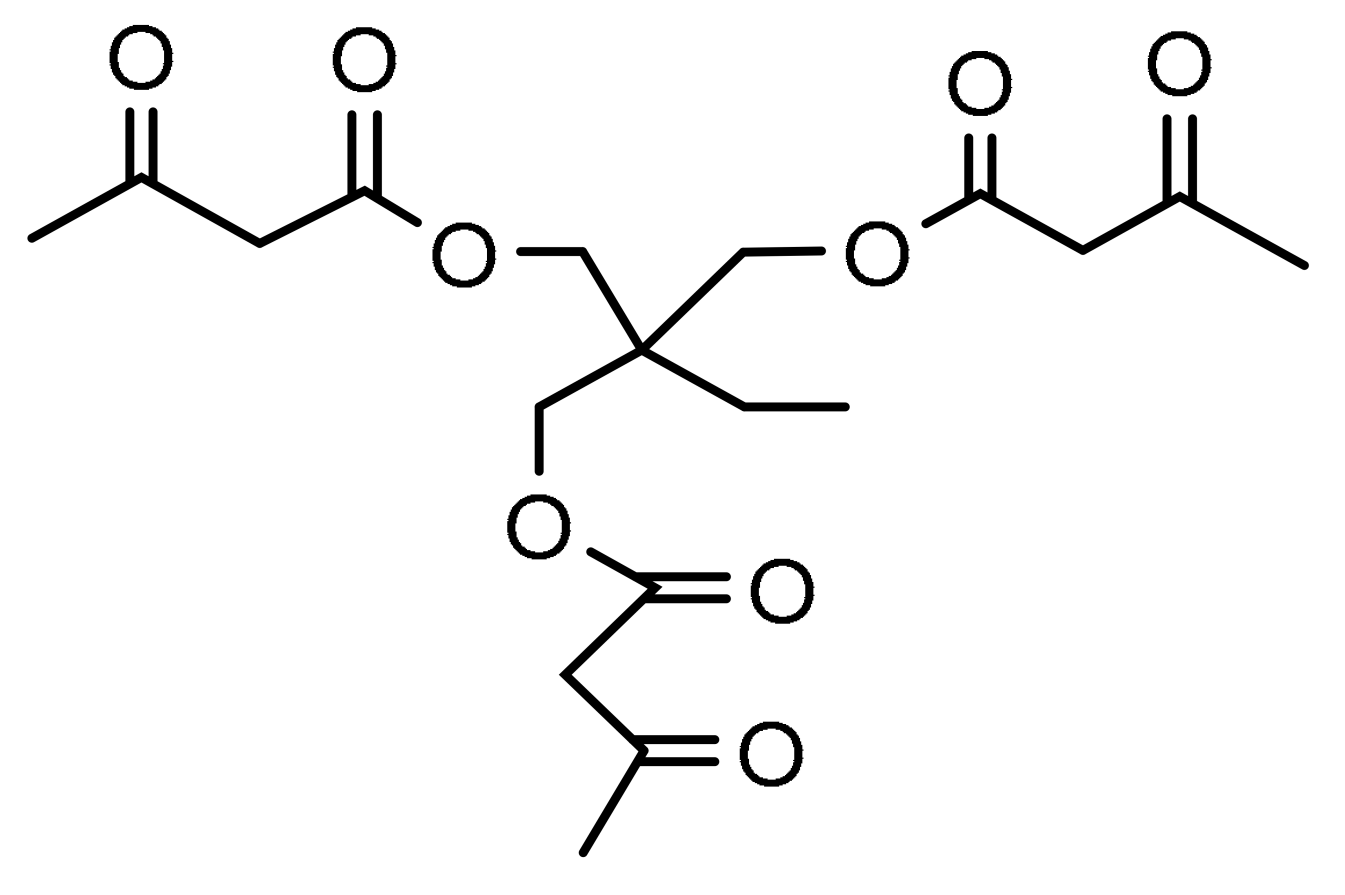	KF
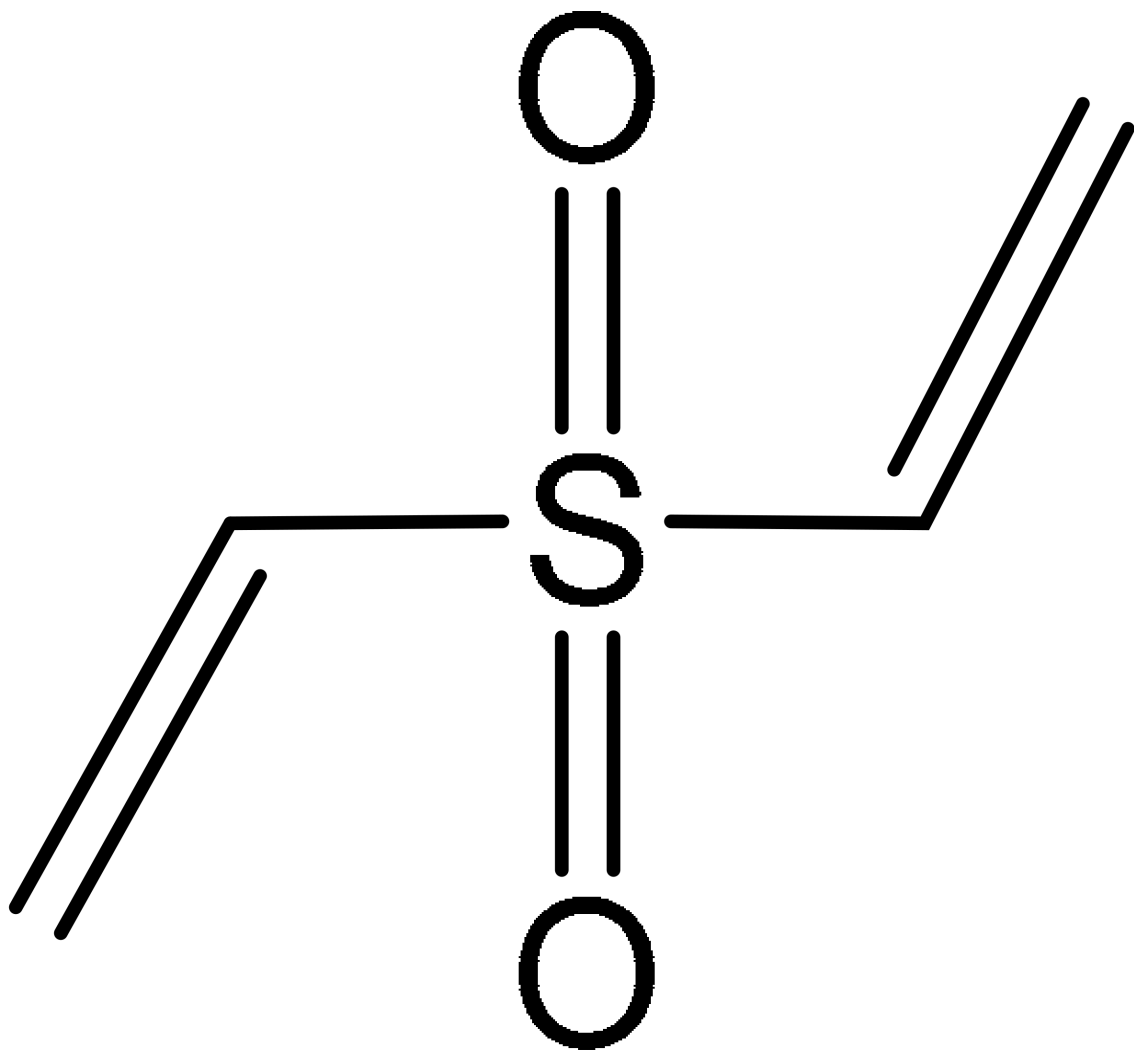	DVS
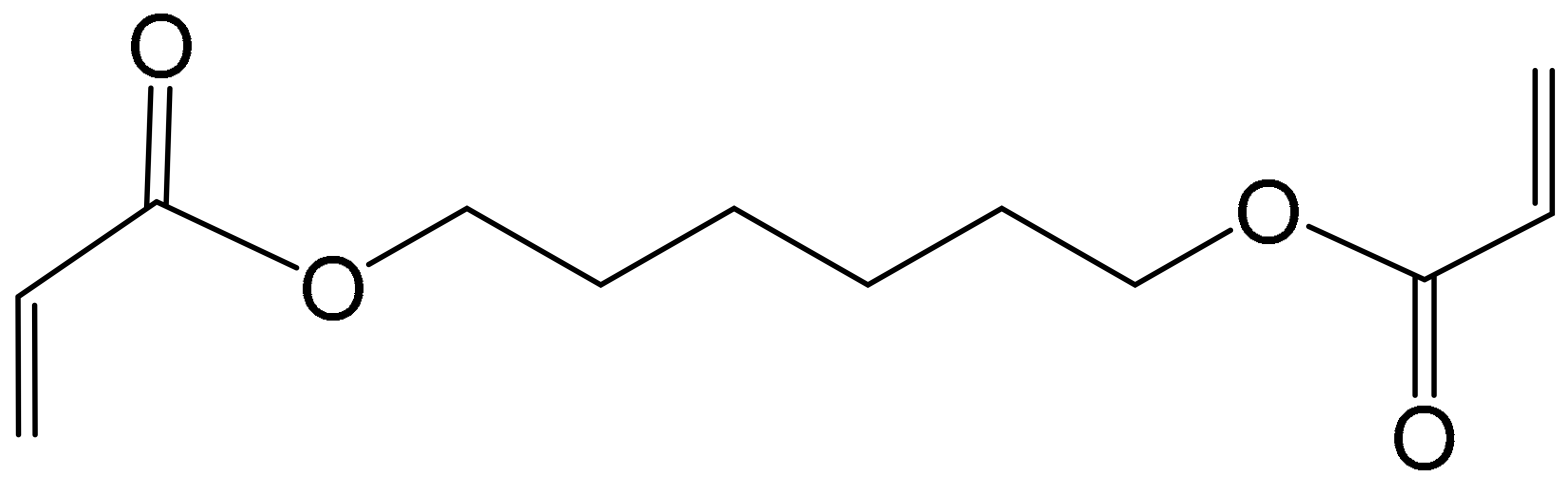	HDDA
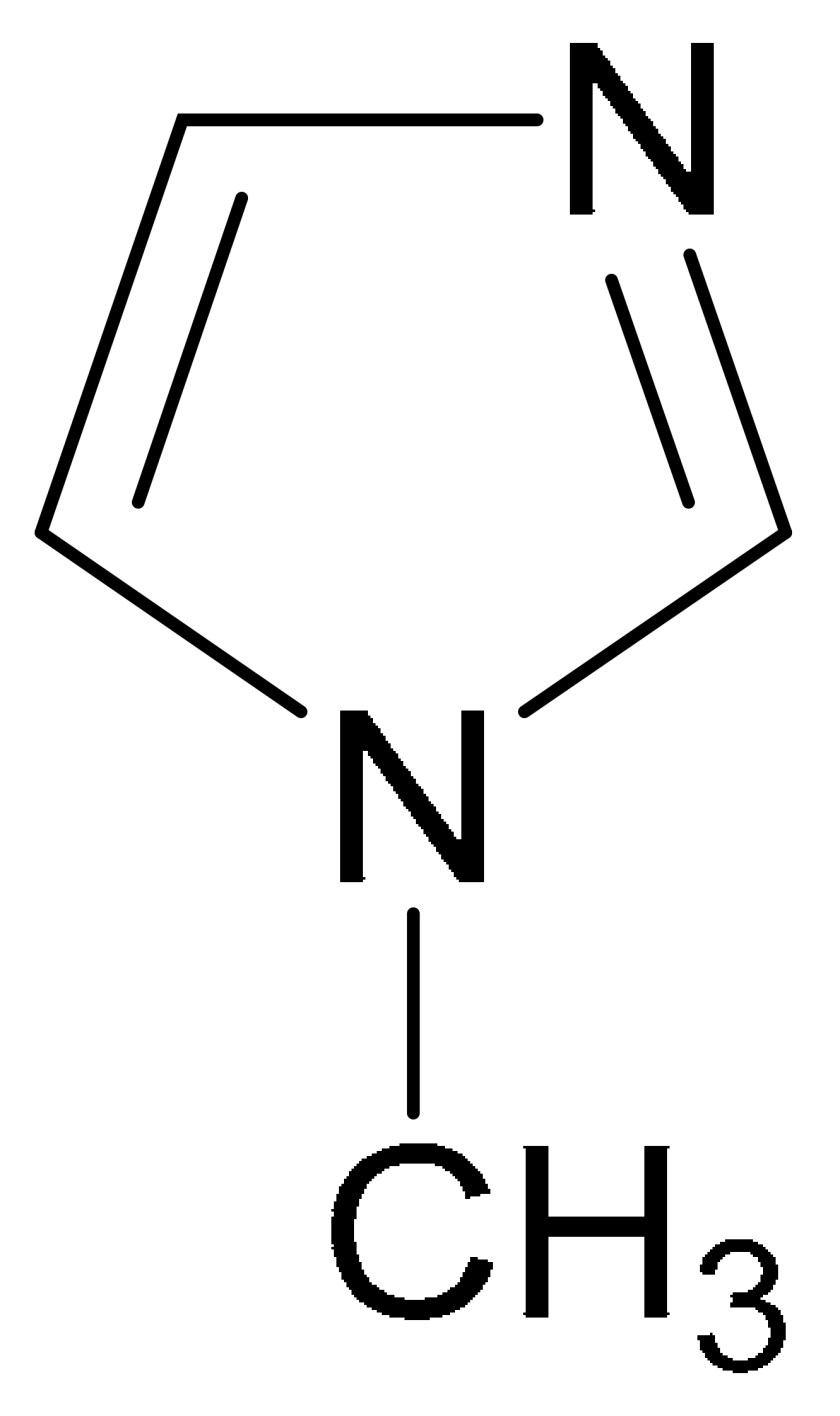	1-MI

**Table 2:** The composition of the dual-curing formulations prepared for the study, assuming *f* = 6 for the acetoacetate KF.

Formulation	KF:DVS:HDDA Molar Ratio	Weight Fractions			
		KF	DVS	HDDA	1-MI
*DVS-HDDA 1-0*	1:1:0	0.51	0.49	0	0.02
*DVS-HDDA 3-1*	4:3:1	0.46	0.33	0.21	0.02
*DVS-HDDA 1-1*	2:1:1	0.42	0.20	0.38	0.02
*DVS-HDDA 1-3*	4:1:3	0.39	0.09	0.52	0.02
*DVS-HDDA 0-1*	1:0:1	0.36	0	0.64	0.02

**Table 3:** The maximum vinyl differential scanning calorimetry (DSC) conversions (*x*) achieved in KF-DVS Michael addition at different reaction temperatures (*T*). The reactions were catalyzed by 0.5% (*w*/*w*) 1-MI. ^a^ KF:DVS molar ratio was 0.68:0.32, ^b^ KF:DVS molar ratio was 0.52:0.48, ^c^ Time to reach a conversion plateau.

Functionality	*T* (°C)	*x* (%)	t (min) ^c^
3 ^a^	35	92	300
	80	98	180
6 ^b^	35	77	240
	80	90	90

**Table 4:** DSC determined parameters of *DVS-HDDA 1-0* used to construct the Venditti-Gillham curves (Equation (2)).

***f***	**3**	**6**
*T_g0_* (K)	195.2	184.8
*T*_*g*∞_ (K)	325.3	443.4
*ΔC*_*p*0_ (J/g·K)	0.619	0.666
*ΔC*_*p*∞_ (J/g·K)	0.396	0.243
*λ*	0.640	0.365

## Supplementary Materials

The following are available online at https://www.mdpi.com/2073-4360/11/9/1408/s1, Figure S1: SEM images of *DVS-HDDA 7-*1 final material.
