# Supplementary material for: Acetoacetate Based Thermosets Prepared by Dual-Michael Addition Reactions
Source: Polymers (Basel). 2019 Aug 27;11(9):1408. doi: 10.3390/polym11091408 (PMC6780194; doi:10.3390/polym11091408)
Supplement: Supplementary file 1 [file polymers-11-01408-s001.pdf]

Supporting information

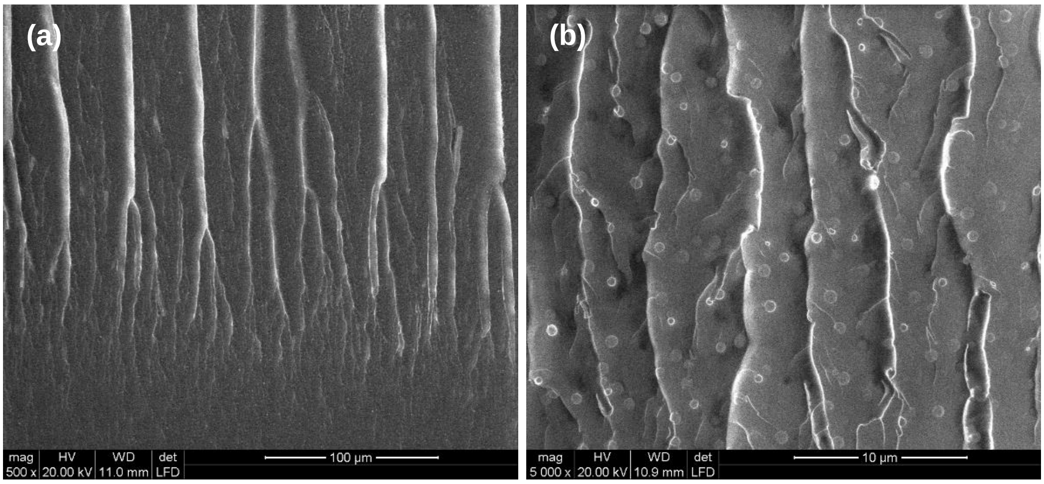

Figure S1. SEM images of *DVS-HDDA 7*-1 final material. At a scale of 100 microns, the sample appears homogeneous **(a)**. When magnified, the phase rich in KF-HDDA is observed as covalently bound spheres within the continuous KF-DVS network **(b)**.
